# Lactation physiokinetics—using advances in technology for a fresh perspective on human milk transfer

**DOI:** 10.3389/fped.2023.1264286

**Published:** 2023-10-16

**Authors:** Jimi Francis, Paul Flynn, Maisha Naowar, Premananda Indic, Darby Dickton

**Affiliations:** ^1^ Integrated Nutrition and Performance Laboratory, Department of Kinesiology, College for Health, Community and Policy, University of Texas at San Antonio, San Antonio, TX, United States; ^2^ Department of Electrical & Computer Engineering, Klesse College of Engineering and Integrated Design, University of Texas at San Antonio, San Antonio, TX, United States; ^3^ Department of Public Health, College for Health, Community and Policy, University of Texas at San Antonio, San Antonio, TX, United States; ^4^ Department of Electrical Engineering, Center for Health Informatics & Analytics (CHIA) University of Texas at Tyler, Tyler, TX, United States; ^5^ Department of Clinical Research, Foundation for Maternal, Infant, and Lactation Knowledge, San Antonio, TX, United States

**Keywords:** breastfeeding, physiokinetics, human milk transfer, oral pressure, biosensor

## Abstract

**Introduction:**

Though the nature of breastfeeding is critical, scant information is available on how the action of the milk transfer from mother to infant is regulated in humans, where the points of dysfunction are, and what can be done to optimize breastfeeding outcomes. While better therapeutic strategies are needed, before they can be devised, a basic scientific understanding of the biomechanical mechanisms that regulate human milk transfer from breast to stomach must first be identified, defined, and understood.

**Methods:**

Combining systems biology and systems medicine into a conceptual framework, using engineering design principles, this work investigates the use of biosensors to characterize human milk flow from the breast to the infant's stomach to identify points of regulation. This exploratory study used this framework to characterize Maternal/Infant Lactation physioKinetics (MILK) utilizing a Biosensor ARray (BAR) as a data collection method.

**Results:**

Participants tolerated the MILKBAR well during data collection. Changes in breast turgor and temperature were significant and related to the volume of milk transferred from the breast. The total milk volume transferred was evaluated in relation to contact force, oral pressure, and jaw movement. Contact force was correlated with milk flow. Oral pressure appears to be a redundant measure and reflective of jaw movements.

**Discussion:**

Nipple and breast turgor, jaw movement, and swallowing were associated with the mass of milk transferred to the infant's stomach. More investigation is needed to better quantify the mass of milk transferred in relation to each variable and understand how each variable regulates milk transfer.

## Introduction

The importance of breastfeeding is well recognized, and the success of breastfeeding promotion in the United States is seen in the increase in initiation rates from 33% in 1975 (1) to 88% in 2019 (2). In many low- and middle-income countries, the weighted prevalence of early initiation of breastfeeding was 52% (3). In European countries, while 56%–97% of infants receive human milk at birth, exclusive breastfeeding rates are declining (4). According to the 2020 Breastfeeding Report Card, (Centers for Disease Control and Prevention (5) there needs to be more progress in developing evidence-based interventions that lead to increased breastfeeding duration (6). The most commonly stated reason for stopping breastfeeding is the maternal perception of inadequate milk supply (7), grounded in low volumes of milk transferred to infants (8). However, the perception of insufficient milk (9) is not a moment in time but a cascade of events. Milk supply is determined by the amount removed from the breast after the initial hormone-driven period of approximately 72 h post-delivery. Breastfeeding is a learned behavior, and for over 50 years in the last century, breastfeeding rates were meager (10), with much of the practical knowledge about breastfeeding lost in the United States as that knowledge was not passed on to many of the young medical practitioners or to new mothers desiring to breastfeed as breastfeeding initiation rates dropped below 33% (11).

Unlike other biologic systems and the fundamental nature of breastfeeding to human existence, scant information is available on how milk flow is established between mother and infant, its paired regulation, and the potential points of dysfunction. Much of the practical information about breastfeeding, such as nuances of positioning and recognition of resolutions, was lost and needed to be recovered. The use of new technology could lead to improved breastfeeding outcomes.

The current understanding of milk transfer does not have a sufficient evidentiary basis for planning interventions and supporting breastfeeding dyads when milk transfer fails. Indeed Lee and Kelleher point out that “*understanding factors that impact lactation and developing methods to assess lactation outcomes before a breastfed infant becomes ill accurately will directly inform the development of therapeutic strategies to improve poor lactation performance*” (12). The technology to resolve this problem is not available as very little is known about the relationships between the maternal and infant inputs that make up this “living” biological secretion of human milk (13). We know that current support techniques do not ensure breastfeeding success (14–16), especially in areas of underserved populations and where lactation professionals are scarce.

The actions exhibited by infants at the breast differ significantly from the skills they use to feed from a bottle. A feeding bottle equipped with a small video recorder and a pressure sensor was used to evaluate tongue movements and oral pressure, with the researchers concluding that jaw motion was correlated with oral pressure (17). Ultrasound imaging has been used extensively to compare different types of artificial nipples in bottle-feeding infants (18–20). Ultrasound has also been used to further understand the infant oral mechanics of the breastfeeding infant, mainly in research settings. Geddes et al., supporting the premise that tongue movements are associated with milk flow into the mouth (21). We theorize that infants who are feeding well, to a great extent, control the flow and volume of milk in some way rather than simply a function of oral pressure changes. Indications of this premise can be seen in numerous ultrasound studies on the importance of the lower portion of the infant’s face in contact with the breast to optimize milk flow (22). The mandible movement of the tongue corresponds with milk flow in breastfeeding infants (23). In another ultrasound study, vacuum strengths were not associated with milk intake but were related to the time spent actively feeding (24). It has been suggested that the absence of milk alters tongue movement (25). More recently, research on tongue kinematics has shown differences in movements during breast- and bottle-feeding using *in vivo* submental ultrasound video clips (26). Douglas and Geddes noted that physiologic approaches are insufficient to ensure successful breastfeeding for many women in the weeks and months post-birth (14).

There has been considerable controversy regarding how infants remove milk from the breast. Despite claims to resolve the dispute through ultrasound imaging and computational simulations (27), an accurate understanding of how the infant facilitates milk removal from the breast remains elusive.

Much biomechanical research on infant feeding has been based on what infants do when bottle feeding. Lang et al., using a specially designed bottle apparatus, found that the feeding patterns of normal infants were more diverse than expected but did suggest that quantification of infant feeding patterns was possible (28). Additional work did allow for the characterization of differences in the maturation of feeding between healthy preterm and full-term infants using the Orometer feeding device (29). However, this line of inquiry has limited application outside the laboratory setting, and the information gathered on oral pressure only provides one measure when attempting to remedy lactation failure in clinical practice.

While the inefficiencies in the system can be complex and perplexing to pinpoint (7, 30), system dysregulation is manifested via malnutrition (31), irrecoverable immune deficiency (32), short- and long-term morbidities (33), and poor health outcomes (34). The problems should be recognized sooner for timely interventions to correct the issues of inadequate milk transfer. Before developing better therapeutic strategies, the physiologic and biomechanics that regulate human milk transfer from breast to stomach must be identified, defined, and understood as interrelated systems. There are hidden interactions between mother and infant, regulated concomitantly, which need to be mapped and characterized to better understand the regulatory points before developing therapeutic strategies. Systems Biology focuses on the complex interactions within biological microcosms aimed at understanding complex biologic processes. Breastfeeding is a biological system that needs scientific investigation to elucidate the biomechanical mechanisms that regulate milk movement from mother to infant. At the same time, it has been suggested that human milk should be considered a biological system (13). Clearly, a new perspective is needed to advance the current understanding of human milk transfer in a way that can lead to an increased number of families meeting their breastfeeding goals. A more comprehensive approach is needed to facilitate the development of strategies to minimize the dysfunction of the human milk transfer system.

Systems biology provides a framework for understanding biology as an interconnected and dynamic system, enabling researchers to explore and manipulate biological processes more comprehensively and interactively. Systems Biology uses four fundamental properties to characterize biologic systems: **system structures** which include physiologic, biochemical, and mechanical components of the system; **system dynamics** of how the process behaves over time and under varying conditions; **control methods** which methodically regulate those processes; and **design methods** which can be used to modulate those processes. Systems medicine aims to transform healthcare by incorporating a holistic understanding of an individual’s biology and environmental factors. Systems Medicine is the application of Systems Biology for **predicting function**, **preventing malfunction**, **personalizing interventions**, and **resolving the problem** with participatory engagement (35, 36). Combining a Systems Biology (37) and Systems Medicine (38) approach to create a new conceptual framework for milk transfer can provide a better understanding of the regulatory mechanisms of human milk transfer from mammary ducts in the breast to the infant’s stomach. Using this framework, pairing biology and medicine creates a multidisciplinary approach that may bring better understanding to the function of breastfeeding physiology and biomechanics by integrating large-scale data. Utilizing engineering design principles (to recognize and define the need, seek systemic causes, and establish baseline parameters to create iterative solutions) can support this new conceptual framework.

While some features of human milk transfer, such as milk flow (39) (maternal side) and swallowing (40) (infant side), have been identified, little is known about regulatory points throughout the Maternal/Infant Lactation physioKinetics (MILK) system. This research study explores the scientific questions about potential regulatory points for milk transfer in the MILK system.

## Materials and methods

The Institutional Review Boards for the University of Texas approved the research protocol. The study population comprised a convenience sample. Mother/infant dyads were recruited from the community through flyers, email list announcements, breastfeeding support groups, and ongoing maternal health studies. Mother/infant dyads were screened for eligibility through a telephone interview with the study team. Inclusion criteria for mothers are (1) an age range of 18–50 years; (2) intention to breastfeed for at least six weeks; and (3) intention to feed directly from the breast. Inclusion criteria for infants are those who (1) were born at 38–42 weeks gestational age, and (2) returned to birth weight by two weeks after delivery with breastfeeding. Exclusion criteria for mothers include (1) maternal age < 18 years; (2) presence of inverted nipples; (3) tape allergy; and (4) history of smoking, which may decrease maternal milk supply. Exclusion criteria for infants are those who were (1) <38 weeks gestational age and (2) diagnosed with ankyloglossia or other congenital anomalies that affect feeding. If the infant cried for 60 s during the sensors’ placement, the session ended, and the sensors were removed as this was considered infant dissent per the research protocol.

The research team completed a health history and demographics form at the telephone interview. Each participant electronically signed a written consent form for herself and her infant. This study collected data from the mother-infant dyad in three fragments: initial data before breastfeeding, active data during the breastfeeding session, and data after breastfeeding. The primary aim of this study was to understand better and explain the complex physiological and anatomical properties of mammary tissue during milk transfer and infant orofacial muscle and bone movement during feeding. Maternal side data included the variables of breast skin temperature, breast turgor, nipple turgor, and maternal weight. Infant variables included temporomandibular joint movement, intra-oral pressure, infant temperature, contact force, and swallowing.

At each observation session prior to feeding, the mother was weighed, nipple diameter and length were measured, and breast turgor and breast temperature were recorded. The nipples were measured in millimeters using digital calipers. Breast turgor was evaluated using a durometer. The breast temperature was measured in degrees Celsius using an infrared thermometer. The mother was settled into a comfortable position. Sensors were applied to the breast for contact force, intraoral pressure, and infants’ nasal airflow. Nasal temperature was measured by a thermistor type (10 kΩ @ 25°C) nasal temperature probe (ADInstruments) connected to a bridge amplifier with noise filtering for fast temperature transient monitoring with an output voltage of 50 mV/°C and a response time of about 200 milliseconds. The probe was placed approximately 1 cm from the infant’s nasal passage during breastfeeding and secured to the mother’s breast using surgical tape.

The infant was measured in centimeters for length and weighed in a clean diaper. Infant weight was measured twice (before and after feeding) in the session, using a Tanita BD-815 U Neonatal / Lactation Baby Scale. The sensors for jaw movement and swallowing were placed on the infant, and the infant was then placed at the mother’s breast. We incorporated the nasal probe with the intention of determining nasal temperature, which served to identify the patterns of inhalation and exhalation. While exhalation leads to a temperature increase, inhalation results in decreased temperature. Data collection commenced as the infant was placed at the breast. Clinical notations were made for infants unlatching from the breast, fussiness, and sensor disruptions. When the infant signaled the completion of the feeding, the sensors continued collecting data for 60 s. The sensors were then removed from the infant. The infant was weighed for the second time. The mother’s after-feeding breast temperature, turgor, and weight were recorded.

Descriptive statistics were used to describe the basic features of the data. Central tendency and dispersion measures were calculated, including mean, median, minimum, maximum, and standard deviation. Multiple logistic regression was used for suggestions about which independent variables influenced the volume of milk transferred. ANOVA statistical tests were performed using Jupiter and SPSS.

## Results

In total, fourteen breastfeeding sessions were recorded. The mean age of the adult participants was 31 years (SD ± 1.8) Overall, 66% of the participants identified as Hispanic with the remaining participants identified as White. All of the participants had a college degree.

The mean infant age was 49 days (SD + 25) of age. The mean mass of milk transferred was 120 g (SD ± 57) per feeding session. The range of the mass of milk transferred was 34 g–222 g.

For this exploratory study, we focused on evaluating the nipple and breast turgor contact force, jaw movement, and oral pressure. There were additional measurements collected of infant respiration (using a respiratory belt or nasal temperature) and swallowing (using hyoid movement).

**Figure 1:**
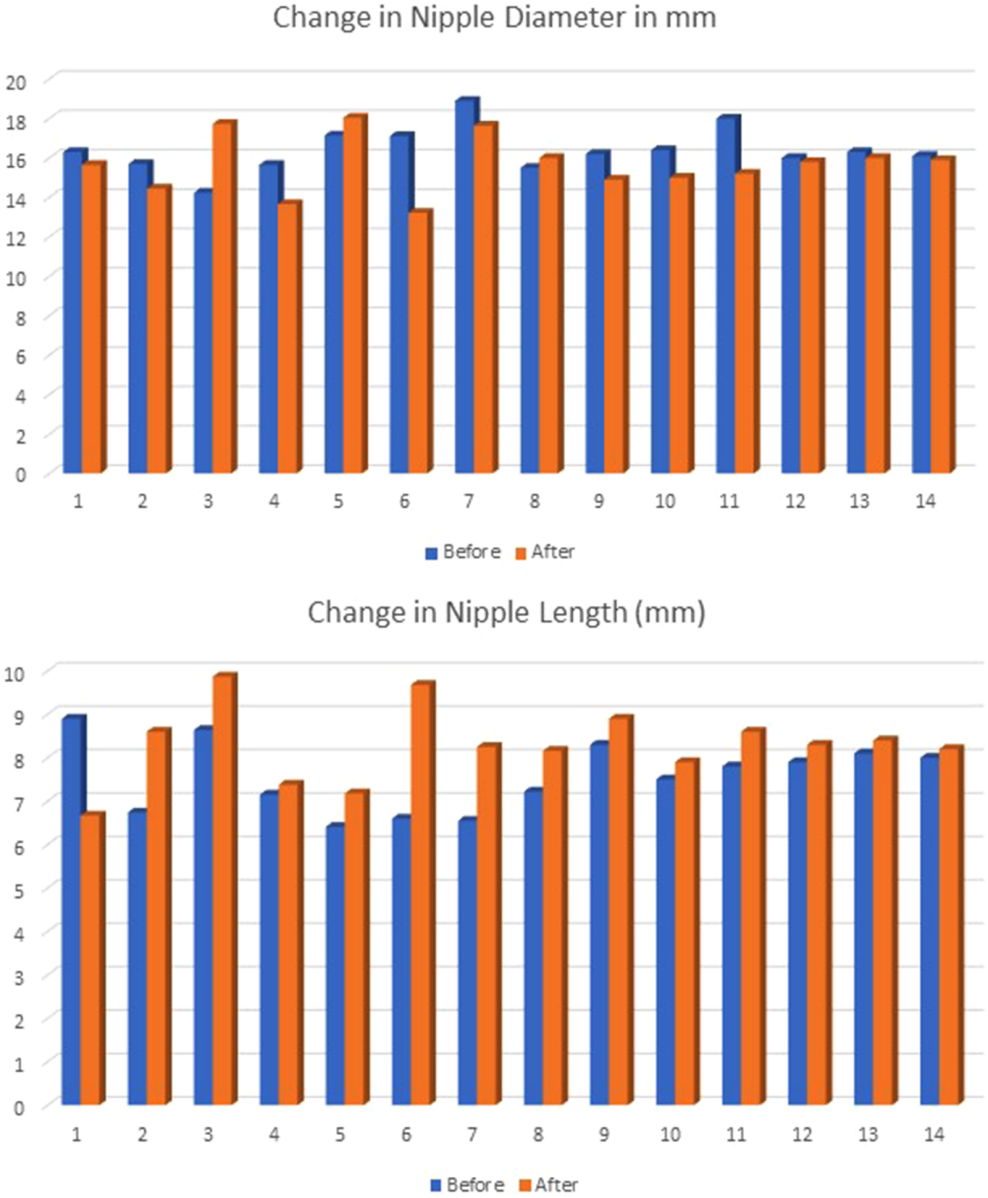
The values for nipple diameter and length were measured in millimeters before and after feeding and were not statistically significant in paired *t*-tests (diameter *p* = 0.171; length *p* = 0.055).

Nipple turgor was evaluated using the change in diameter and length between pre- and after-feeding measurements. The average nipple diameter before feeding was 16.4 mm and 15.7 mm after feeding. The average nipple length before feeding was 7.6 mm and 8.2 mm after feeding. Nipple diameter was typically slightly less after feeding, while nipple length was typically longer after providing no statistical difference, as shown in Figure 1. The mean change in nipple diameter was −0.74 (SD ± 1.75). The mean change in nipple length was 0.67 mm (SD ± 1.19).

**Figure 2:**
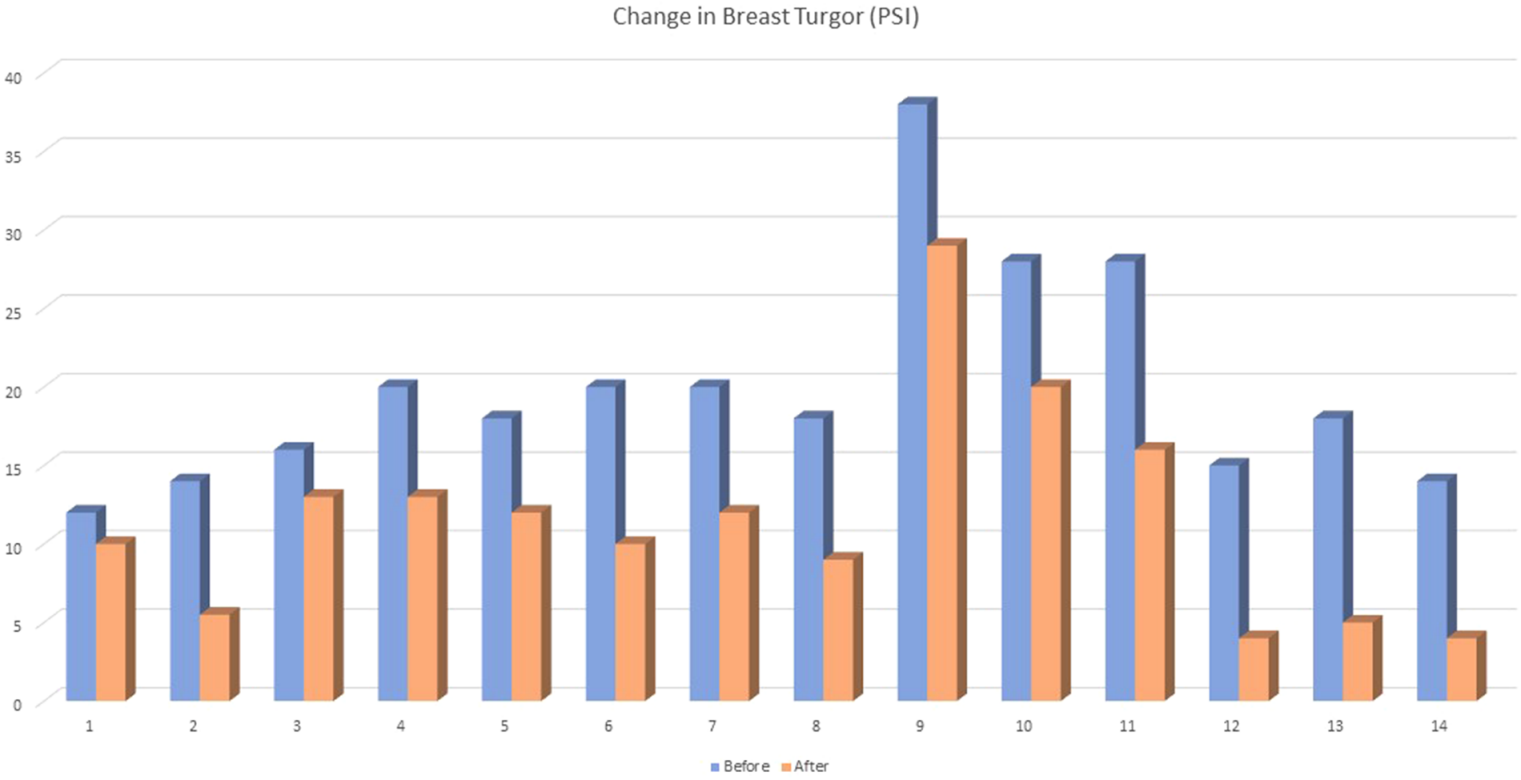
Breast turgor is measured in pounds per square inch before and after breastfeeding.

Breast turgor was measured using a type “OO” durometer, which is recognized as an accurate and reliable tool to quantify hardness on various parts of the human body (41). This type of durometer is also recommended for applications involving human skin. tissue (42). The durometer was placed at the area that was 3 cm from the nipples in the 6 o’clock position. Higher measurements represented increased turgor. The mean for breast turgor before breastfeeding was 19.9 pounds per square inch (psi) (SD ± 7.02), ranging from 12 to 38. The mean after breastfeeding was 11.6 psi (SD ± 6.82), with a range from 4 to 29. The before and after differences shown in Figure 2 were statistically significant using paired t-tests with *p* < 0.001.

The volume of milk transferred to infants during feeding was calculated such that mass/density Mass = W(after)-W(before) in grams. Mass was divided by 1.25 g/ml (density of human milk) to get the volume in milliliters. The mean mass of milk transferred to the infant stomach was 119.5 g (SD ± 53) per feeding, ranging from 34 g–222 g. The average time of feeding was 10.5 minutes. The average amount of milk transferred during feeding was 117 ml, ranging from 34 ml to 224 ml. The mean rate of milk transfer was 10.6 ml/minute.

Descriptive statistics were calculated for contact force, Oral Pressure, and jaw movement. Contact force was measured using a Millar pressure sensor, adapted to measure the contact pressure of the infant’s chin against the mother’s breast by converting mechanical force into a voltage signal. Intra-oral pressure was measured using a Millar catheter positioned and attached to the breast of the mother, with the tip of the catheter protruding approximately 3 mm past the tip of the nipple before placing the infant at the breast. Jaw movement was measured using a piezoelectric film and a data acquisition device. Six feeding sessions had complete data needed for the descriptive statistics, as shown in Table 1.

**Table 1:** Summary of the maximum, minimum, and range for contact force, oral pressure, and jaw movement.

	Contact Force (mN)			Oral pressure (mmHg)			Jaw Movement (mV)		
Session	Max.	Min.	Range	Max.	Min.	Range	Max.	Min.	Range
1	80.14	78.31	1.83	−12.68	−188.73	176.04	−6.1	−11.1	5.0
2	79.36	79.32	0.04	−9.5	−333.07	323.56	3.8	−4.4	8.1
3	79.22	79.17	0.05	−16.42	−217.71	201.29	6.5	−7.8	14.3
4	85.92	81.1	4.83	14.74	−162.52	177.25	9.3	−17.8	27.1
5	82.4	78.81	3.59	−19.59	−216.81	197.22	−0.1	−17.3	17.2
6	79.39	79.16	0.23	−18.75	−197.27	178.52	14.8	−14.3	29.1

**Figure 3:**
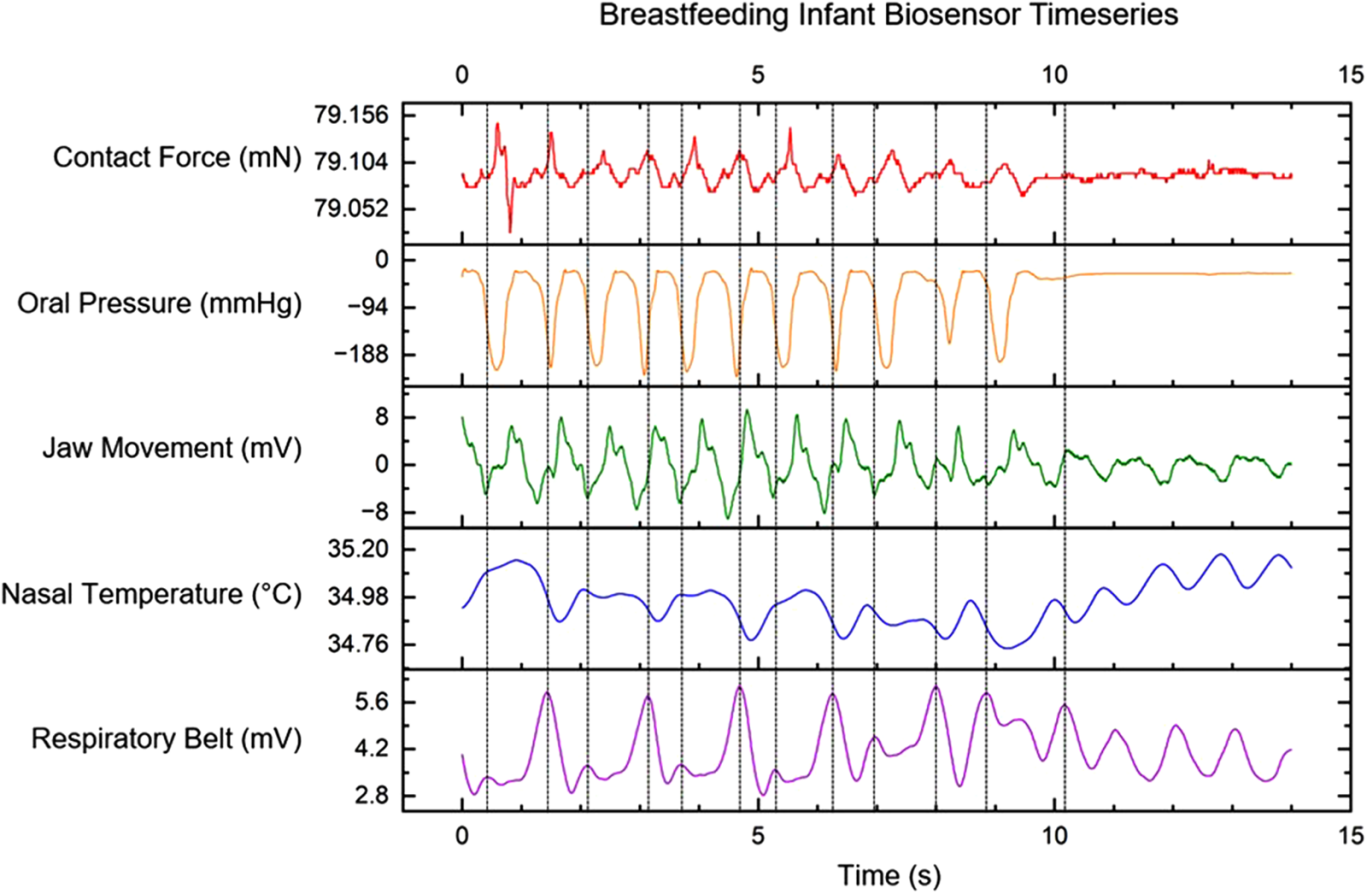
Multi-sensor time-series data for contact force between infant chin and breast, intraoral pressure from MEMs catheter, the signal from PVDF film for sensing jaw movement, air temperature measurements using a nasal cannula, and a signal from the respiratory belt. Vertical dashed lines indicate infant swallows observed by the clinician.

Coherence was demonstrated between the time period of biomechanical action of oral pressure and contact force with jaw movement. ANOVA was performed for the biomechanicl time period between oral pressure and jaw movement, as well as between contact force and jaw movement. Results from ANOVA showed no statistical differrence between all sensor channels (*p*-value < 0.05. This significance indicates a synchronicity of the actions. A rhythmic contact force of approximately 0.1 milliNewtons is maintained between the infant’s chin and the breast. In the representative example seen in Figure 3, the pressure within the infant’s oral cavity fluctuates between the latching pressure of −15 mmHg and −190 mmHg. During active feeding, the jaw movement sensor records regular oscillations of ±8 mV. The nasal temperature sensor channel is an indirect measure of infant respiration. The respiratory belt measures the expansion and contraction of the infant’s diaphragm. Swallows were denoted by the clinician observing the infant during feeding. A correlation was seen with the magnitude and changes in amplitude to the mass of milk transferred to the infant.

The Pharynx is a shared anatomic pathway for both swallowing and breathing; however, these two activities are mutually exclusive. How infant’s co-ordinate the continuous reconfiguration of swallowing and breathing at the same time is still unknown. We incorporated the nasal probe with the intention of determining nasal temperature, which served to identify the patterns of inhalation and exhalation. While exhalation leads to a temperature increase, inhalation results in decreased temperature. The mechanism of nasal temperature regulation which is associated with respiration is exclusive to contact force generation or oral pressure.

To evaluate correlations between Contact Force, Oral Pressure, and Jaw Movements, we estimated the phase of the contact force, oral pressure, and jaw movement using Hilbert transform (43) and explored any phase locking between the signals by calculating its phase difference (44) with unwrapped phase.

**Figure 4:**
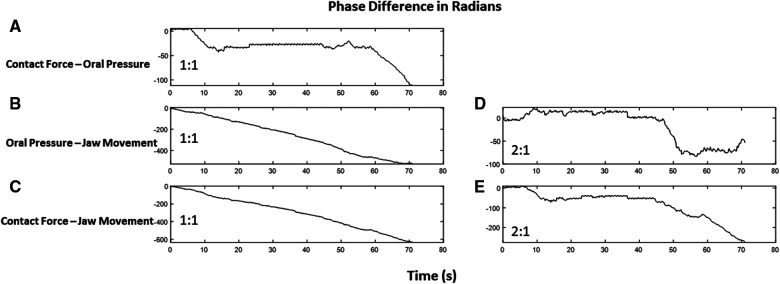
The phase difference between time series in radians with respect to time in seconds. The slope of line zero is an indication of locking. Contact force and oral pressure have a locking of 1:1 as the slope of the jittery phase difference remains zero at several time points (**A**), whereas phase difference linearly changes for oral pressure and jaw movement (**B**) as well as for contact force and jaw movement (**C**). Interestingly, these time series exhibit a 2:1 phase locking, as depicted in (**D**) and (**E**).

With the infant’s age variation, we observed a 1:1 phase-locking phenomenon between contact force and oral pressure. Additionally, a clear indication of 2:1 phase locking between jaw movement with oral pressure and contact force was seen and is shown in Figure 4. The peak-to-peak difference in an infant’s jaw movement is twice the oscillation in context to oral pressure or contact pressure. However, older infants exhibit 1:1 phase locking between time series.

## Discussion

While the sample was small, the percentage of Hispanics in the study is representative of the local population, as 67% of the population is identified as Hispanic with 23% identified as White. Additional racial diversity will be included in future study.

The volume of milk intake was appropriate at each feeding session considering the length of time from the prior feeding session, the size, and age of the infant.

Nipple turgor, as measured by nipple length and diameter, was similar to those values that have been previously reported (45). The values, in this study, for before and after breastfeeding were not statistically significant and were not visually different after the baby disconnected from the nipple. Any change in nipple lengthening or diameter increases of the nipple were transitory and may be explained by the contraction of the muscle cells surrounding the nipple itself as occurs upon nipple stimulation, such as when the nipples are exposed to cold, becoming evert. The relationship to breast turgor, if any, needs further evaluation.

Breast turgor refers to the elasticity and firmness of the breast tissue and was used in this study to assess changes in fluid balance. Before the breastfeeding sessions, the breast tissue appeared firm yet elastic. The change seen in breast turgor indicates a fluid shift. Further analysis is needed to evaluate any relationship between change in breast turgor, maternal weight, and mass of milk transferred.

To initiate feeding at the breast, the infant’s mouth is open wide, encompassing a significant portion of the areola and the nipple with the chin in firm contact (contact force) with the breast. This study’s initial contact force is more robust and lessens with each successive let-down reflex. Contact force variation drops to zero by the end of the feeding. In this study, we observed that contact force was essential to milk ejection, particularly at the beginning of active feeding.

Breastfeeding includes active feeding and quiescent attachment. There are several muscles involved during both active feeding and quiescent attachment. The tongue plays a crucial role in breastfeeding. The infant’s tongue moves in a wave-like motion, pressing against the breast and creating the movement necessary to elicit milk flow into the mouth. The coordinated movement of the tongue helps in moving milk from the breast to the mouth. The lips and chin location facilitate the maintenance the latch, while the jaw movement enables the anterior tongue to move as a lever to compress the teat formed by the nipple/areolar complex to control volume of the flow. The jaw and neck muscles are responsible for the undulating movement of the mid-tongue, enabling the baby to create negative pressure to keep the milk flowing. The jaw muscles coordinate with the tongue to create a rhythmic feeding motion. Various facial muscles are engaged during breastfeeding as well. The muscles around the mouth, including the orbicularis oris muscle, help form a secure seal around the breast to maintain suction. These muscles work together to ensure a proper latch and prevent milk leakage. The muscles in the cheeks, such as the buccinator muscles, play a supportive role during breastfeeding to hold the milk in the mouth until the airway is protected.

Fifty pairs of muscles and six cranial nerves working together for human beings to swallow (46). The buccal phase of swallowing is voluntary. The tip of the tongue encircles the nipple/areolar complex, compressing it against the alveolar ridge of the hard palate, while the posterior tongue drops to create a space for milk to be held until the air way is protected. The tongue surface moves upward, gradually expanding the area and squeezing the liquid bolus back along the palate and into the pharynx. It is important to note that the coordination and strength of these muscles develop and improve as the infant grows and gains feeding experience (47). Also, breastfeeding helps develop oral motor skills, muscles used in speech and swallowing later in life. Neurological maturation associated with experiential learning facilitates the transformation in feeding patterns of infants (48).

With respiration, on inspiration our findings demonstrated lower voltage reading, and on expiration, a higher voltage was demonstrated. The respiration rate was approximately 60 breaths per min using the nasal temperature sensor and the respiratory belt. Compared to the quiescent attachment, we see truncated peaks, evidence of the infant’s breath being held to swallow. There is an apparent synchronization between diaphragm expansion and observed swallows.

In this study, we observed that once the infant latched to the nipple-areolar complex, they use contact force to elicit milk ejection. They use their tongue, jaw, and facial muscles to manage milk flow. In active feeding, the tongue, palate, and cheeks trap the milk flowing into the mouth to create a bolus of milk, while the epiglottis closes over the larynx to protect the airway. The milk is then swallowed in coordinated pauses of breathing. Breastfeeding is a coordination of milk flow and infants’ control of positive and negative pressure.

During active feeding, oral pressure is directly correlated with the movement of the jaw. During quiescent attachment, the oral pressure returns to a steady negative value near the latching pressure. Our findings show that negative oral pressure peaked when the jaw was most extended, indicating that changes in oral pressure can be seen with the movement of the jaw. The negative oral pressure decreased as the jaw moved toward a neutral position, while maintaining latch pressure. Peak vacuum (−152 ± 38 mmHg) occurred when the jaw was in the lowest position. Positive pressure occurs when the jaw elevates, lips are sealed, and the mid-blade of the tongue elevates to the hard palate. Negative pressure occurs when the jaw drops, moving the tongue away from the hard palate, and the lips remain sealed. The amplitude of the jaw movement sensor drops by more than half to less than ±4 mV. The frequency of jaw motion is also reduced during active feeding.

Analysis of the pilot study data set for connections between the physiologic parameters found and the actual quantity/volume of milk transferred suggest relationships between some measurables and mass of milk transferred. For example, there appears to be a positive trend between the percent change of breast turgor and mass of milk transferred. Additionally, we can observe a similar positive trend between the ‘active feeding/nutritive suckling” time and the mass of milk transferred. The “active feeding” time can be easily calculated by analyzing the contact force, oral pressure, and jaw movement sensor channels. By setting threshold values for frequency and amplitude that correspond to active latch and feeding as observed by a clinician, an estimate for the length of time of active milk transfer. These initial findings are suggestive of correlations between the amount of milk transferred and specific measures from biosensors, but the limited size of the pilot study dataset prevents a final conclusion on these observations.

Utilizing Systems Medicine to apply Systems Biology to the human milk transfer systems has enabled the identification and characterization of aspects of the biomechanical and physiologic components of the Maternal/Infant Lactation physioKinetics (MILK) system. With this framework, we can begin to systematically evaluate the dynamics of milk movement from the lactating breast to the infant stomach for predicting function, preventing malfunction, personalizing interventions, and resolving the problem with participatory engagement of the mother and infant.

Despite the small sample size, we can discern and quantify that nipple and breast turgor, jaw movement and swallowing are associated with the mass of milk transferred to the infant stomach. More investigation is needed to better quantify mass of milk transferred and understand how the process behaves over time and under varying conditions such as infants who are not gaining weight appropriately or pain for the mother when breastfeeding.

Based on our observations and data analysis, we conclude that the negative change in intraoral pressure is a function of jaw movement rather than the infant applying negative pressure to remove milk. Both active and quiescent feeding movements are a coordination of mandibular protrusion and retrusion. This jaw motion appears to be a regulatory or driving mechanism behind both the contact force and oral pressure readings. Both analysis methods strongly support a quantitative coherence between jaw movement with oral pressure and contact force. Additional research is needed to make further conclusions about the regulatory mechanisms of the Maternal/Infant Lactation physioKinetics (MILK) system.

## Data Availability

The raw data supporting the conclusions of this article will be made available by the authors, without undue reservation.
